# Editorial: Emergency in Psychiatry—The Various Facets of Behavioral Emergencies, Crises, and Suicidality

**DOI:** 10.3389/fpsyt.2021.668154

**Published:** 2021-03-19

**Authors:** Johannes M. Hennings, Dagmar I. Keller, Ksenija Slankamenac, Thomas C. Wetter

**Affiliations:** ^1^Department of Dialectical Behavioral Therapy, kbo-Isar-Amper-Klinikum Munich-East, Munich, Germany; ^2^Emergency Department, University Hospital Zurich, Zurich, Switzerland; ^3^Department of Psychiatry and Psychotherapy, University of Regensburg, Regensburg, Germany

**Keywords:** psychiatry, suicidality, crisis, self-mutilation, emergency, sleep, borderline personality disorder, adolescent psychiatry

Psychiatric emergencies occur every day in various presentations and in various clinical settings. Not only social workers, relatives and teachers can be stunned, overwhelmed and helpless in these situations but also health care professionals are. Social workers and emergency service providers dealing with these highly stressful situations may themselves be traumatized by this confrontation and may suffer significant psychological damage (1–3). In adult as well as in child and adolescent mental health emergencies, self-mutilating and suicidal behavior are within the most frequent challenges as documented in the analyses of Franzen et al. and Slankamenac et al. within this issue. Suicidal crises may trigger feelings of anxiety and anger in those who treat these patients (4). In addition, the acute and long-term treatment of these patients is still demanding–especially in the case of chronic suicidality that often exists in individuals repeatedly being admitted to emergency units with self-mutilation, intoxications, or suicide attempts (5, 6). Unfortunately, the therapist's fear that a patient may commit suicide can threaten clinical judgment, contribute to problems in therapy and may seriously impede the therapist's ability to deal effectively with the danger of suicide (7).

In this issue, we specifically compile articles that focus on interventions and skills that may help individual professionals as well as teams dealing with such emergencies in psychiatry.

Bolsinger et al. stress the importance of a good therapeutic relationship for this endeavor showing special attributes and caveats in an acute psychiatric setting. Across countries, crisis lines have become an inherent part of the crisis management and prevention of suicides. Hoffberg et al. found evidence for the effectiveness of crisis lines but observe that there is still an important gap in the evaluation of this mean of help. Including a medical developmental and systemic perspective, Guedj et al. propose a comprehensive and operational model for the management of adolescents with behavioral problems in an emergency department.

Repetitive transcranial magnetic stimulation has also been discussed in the context of its potential ability to rapidly reduce suicidality (8, 9). Within this special issue, a comprehensive overview (Abdelnaim et al.) of these emergent advances for suicidality in depressed patients is presented. Further, a suggested link between suicidality and sleep disturbances in the context of post-traumatic stress disorder (PTSD) as well as its possible therapeutic implications are scrutinized in a thorough literature overview (Weber et al.). Heterogeneous study approaches and diverse outcome parameters hinder a direct comparison of studies examining sleep disturbances, suicidality, and PTSD. However, sleep problems as still underestimated target symptoms may provide preventive strategies with respect to suicidality.

Finally, yet importantly, patients with personality disorders, especially borderline personality disorders (BPD), deserve specific attention as they are typically afflicted with frequent crises including states of acute and chronic suicidality leading to highly frequent usage of psychiatric as well as general emergency services (Slankamenac et al.). Early and focused interventions being of utmost importance in these cases, we discuss promising therapeutic approaches (Hennings) specifically addressing recurrent suicidality in BPD.

## Author Contributions

JH wrote the first draft of the manuscript. All authors contributed to manuscript revision, read, and approved the submitted version.

## Conflict of Interest

The authors declare that the research was conducted in the absence of any commercial or financial relationships that could be construed as a potential conflict of interest.
